# On-farm *Campylobacter* and *Escherichia coli* in commercial broiler chickens: Re-used bedding does not influence *Campylobacter* emergence and levels across sequential farming cycles

**DOI:** 10.3382/ps/pew003

**Published:** 2016-02-16

**Authors:** H. N. Chinivasagam, W. Estella, H. Rodrigues, D. G. Mayer, C. Weyand, T. Tran, A. Onysk, I. Diallo

**Affiliations:** * EcoSciences Precinct, Department of Agriculture and Fisheries, Dutton Park 4102, Queensland, Australia; † Biosecurity Sciences Laboratory, Health and Food Sciences Precinct, Department of Agriculture and Fisheries, PO Box 156 Archerfield BC 4108, Queensland, Australia

**Keywords:** *Campylobacter*, *E. coli*, litter, ceca, chickens

## Abstract

Limitations in quality bedding material have resulted in the growing need to re-use litter during broiler farming in some countries, which can be of concern from a food-safety perspective. The aim of this study was to compare the *Campylobacter* levels in ceca and litter across three litter treatments under commercial farming conditions. The litter treatments were (a) the use of new litter after each farming cycle; (b) an Australian partial litter re-use practice; and (c) a full litter re-use practice. The study was carried out on two farms over two years (Farm 1, from 2009–2010 and Farm 2, from 2010–2011), across three sheds (35,000 to 40,000 chickens/shed) on each farm, adopting three different litter treatments across six commercial cycles. A random sampling design was adopted to test litter and ceca for *Campylobacter* and *Escherichia coli*, prior to commercial first thin-out and final pick-up. *Campylobacter* levels varied little across litter practices and farming cycles on each farm and were in the range of log 8.0–9.0 CFU/g in ceca and log 4.0–6.0 MPN/g for litter. Similarly the *E. coli* in ceca were ∼log 7.0 CFU/g. At first thin-out and final pick-up, the statistical analysis for both litter and ceca showed that the three-way interaction (treatments by farms by times) was highly significant (*P* < 0.01), indicating that the patterns of *Campylobacter* emergence/presence across time vary between the farms, cycles and pickups. The emergence and levels of both organisms were not influenced by litter treatments across the six farming cycles on both farms. Either *C. jejuni* or *C. coli* could be the dominant species across litter and ceca, and this phenomenon could not be attributed to specific litter treatments. Irrespective of the litter treatments in place, cycle 2 on Farm 2 remained *Campylobacter*-free. These outcomes suggest that litter treatments did not directly influence the time of emergence and levels of *Campylobacter* and *E. coli* during commercial farming.

## INTRODUCTION

*Campylobacter* is the common cause of human infectious intestinal disease, mostly in temperate countries (Alter and Scherer, 2006), and is closely associated with poultry and contaminated poultry meat (Wassenaar, 2011). The on-farm management of *Campylobacter* continues to remain a challenge. Flocks with a higher prevalence of *Campylobacter* can have an average of log 5.3 colony forming units (**CFU**) per carcass (and maximum of log 8.0 CFU per carcass) (Allen et al., 2007); thus, they can be of food-safety concern. *C. jejuni* is the most common species associated with human illness and has evolved to preferentially colonize the chicken gut (Snelling et al., 2005). The ecology and the epidemiology of *Campylobacter* in broiler flocks is complex (Hermans et al., 2012). The biology of *Campylobacter* differs from other zoonotic pathogens (such as *Salmonella*), and there exists a need for a better understanding of *Campylobacter* physiology and survival mechanisms in poultry (Ingmer, 2011). These aspects can be directly impacted by both the farming environment and practices adopted across various countries.

The expansion of the poultry industry, along with the scale of production, demands continuous supplies of large volumes of quality bedding material (e.g., wood shavings). There are also related environmental challenges linked to the disposal of bedding after a relatively short poultry farming cycle of approximately 50 d or less where used litter cannot remain on-farm due to biosecurity requirements. These demands have contributed to litter re-use in countries such as Australia and the United States. The emerging practice of re-using bedding (or litter) may have an impact on key food-safety pathogens such as *Campylobacter*. In the US, litter can be re-used for periods lasting for up to 1 to 2 years or more before a full clean-out of litter from a shed (or barn) occurs (Payne et al., 2006; LSU Ag Center, 2011). In Australia, litter can remain in a shed for more than a single farming cycle and up to several cycles (Chinivasagam, 2009). Thus, the prospect of safely re-using litter can contribute towards alleviating food-safety concerns due to the ongoing challenge of managing *Campylobacter* during commercial farming.

On-farm biosecurity during poultry farming is an integral part of farming practice targeting the management of pathogens. Thus, studies have looked at possible biosecurity-based interventions and strategies to reduce on-farm *Campylobacter* (Newell et al., 2011), though it is reported that simple measures do not have a significant influence on the *Campylobacter* status of the flock (Nather et al., 2009). Thus, common hygienic measures that have substantially reduced *Salmonella* have not been effective against *Campylobacter* (Ingmer, 2011). An understanding of the on-farm microbial ecology of pathogens such as *Campylobacter* (Jaykus, 2003) is a key factor to the acceptance of litter re-use, which can be viewed as a concern during commercial farming.

The cecum is the main source for *Campylobacter* colonization (Van Deun et al., 2008), and intestinal microbiota perform an important role in controlling enteric bacterial pathogens (Chambers and Gong, 2011). An Australian study (Torok et al., 2009) has shown that the cecal microbiota of chickens raised on reused litter was significantly (*P* < 0.05) different from that of chickens raised on any of the other litter materials. This study has also shown the influence of bird age with cecal microbial communities (Torok et al., 2009). Chickens are known to ingest litter (which also plays a role in digestion) (Hetland et al., 2003), and studies have demonstrated a significant consumption of litter among broilers from the floor (Svihus et al., 2009). There could be a relationship between the litter practices adopted and the *Campylobacter* numbers in the cecum. *Campylobacter* has shown to have a low infectious dose (approximately 500 organisms) (Robinson, 1981), and thus the management of *Campylobacter* numbers (in ceca) on-farm can contribute to enhanced product safety.

The main aim of the current study was to assess the levels of *Campylobacter* (and *E. coli*) in both litter and ceca in the presence of litter re-use (and without) during normal commercial farming. Three different litter treatments; the conventional practice of cleaning litter after each farming cycle (and using new bedding), the Australian practice of partial re-use (with a mix of used and new bedding) and a full litter re-use practice were studied. In order to assess the impact over time, the three litter treatments were assessed across six sequential farming cycles on two farms.

## MATERIALS AND METHODS

### Farm Sampling

Two separate commercial farms, situated close to Brisbane, Queensland (Australia) with a history of litter re-use (over 20 years) were selected. Farm 1 (**F1**) had 4 sheds with approximately 35,000 chickens per shed, and Farm 2 (**F2**) had 8 sheds with approximately 40,000 chickens per shed. Only three sheds from each farm were included in this study. The farms were adjacent to each other and were managed by a single company. All normal farming practices, including diet, were according to standard industry practice for meat-chicken rearing, as the trial was carried out under commercial farming conditions. The details of the Australian litter re-use practice adopted by commercial farms is described in Chinivasagam et al. (2012), and the litter management practices that occur between farming cycles are described in Chinivasagam (2009).

Animal ethics approval was obtained for the entire experimental study from the Department of Agriculture and Fisheries (Queensland, Australia) animal ethics committee prior to the trial. Prior to the commencement of the trial, three adjacent sheds from each farm underwent a full clean-out of litter, and that cycle (time taken from chick placement to final pick-up) was designated as cycle 1 (**Cy1**); hence all three sheds had similar litter treatments during Cy1. From cycle 2 (**Cy2**) onwards, each shed on both farms had different litter treatments. The litter treatments adopted were designated as *“New”* (**N**), *“Partial re-use”* (**P**) and *“Full Re-use”* (**F**); the conditions adopted on-farm during the trial are described in Table 1. The duration of the six sequential farming cycles were: F1, 23/03/2009 – 13/04/2010 and F2, 29/09/2010 – 09/09/2011.

**Table 1.** Litter treatments and conditions adopted across six sequential farming cycles within the three test sheds on Farm 1^#^ and Farm 2.

	Litter Treatments Adopted on F1 and F2 During the Study		
	New Shed 1	^##^Partial Re-use Shed 2	Full Re-use Shed 3
Nature of bedding	(1) Always new.	(1) New and old.	(1) Always old.
in sheds during the study on F1 and F2	(2) Litter removed after each cycle and new bedding placed.	(2) Litter from brooder moved to grow-out and new bedding placed; litter from grow-out removed from shed	(2) Bedding never removed but re-used across whole shed i.e., brooder and grow-out
Litter management between cycles during the study on F1 and F2 (all sheds cleaned sanitized between cycles, sprayed for insects)	New bedding placed across whole shed	Litter from brooder heaped into pile at grow-out end for ∼5 to 6 d and then spread at grow-out end. Clumps removed.	(1) Farm 1: Litter heaped across entire length of shed for 5 d and spread.(2) Farm 2: Litter aerated mechanically, clumps removed and spread.
Biosecurity measures during cycleson F1 and F2			
The use of footbaths with disinfectant and protective clothing on entry of authorized personnel			

^#^Sixth cycle was run as free-range practice (Free Range Egg & Poultry Australia - FREPA) due to conversion of that farm to free-range.

^##^Described in (Chinivasagam et al., 2012 and Chinivasagam 2009).

### Collection of Litter and Ceca Samples

During each cycle both litter (**L**) and chickens’ ceca (**C**) were sampled from the three sheds on both farms as follows: (a) at ∼7 days into the growth cycle, litter only, (data not shown); (b) just before first pick-up (thinning), litter and chickens; (c) just before final pick-up, litter and chickens. Pick-ups were commercial pick-up days. Litter was collected as in Chinivasagam et al. (2012) to a depth of 40 cm over an area of 400 cm^2^ using a specially designed stainless steel sampler. The sheds were around 100 m long with 39 (F1) and 33 (F2) bays (structure supports along the shed), respectively, which were used as markers for random number allocation (and sample collection) across the shed. Each shed was categorized into four main sections, i.e., two in the brooder area (front-end, 1 and 2) and two in the grow-out area (back-end, 3 and 4).

Sample collection was based on the location of bays, drinkers and feeder as markers for random number allocation for sampling spots. Initially sixteen bays were selected using random number (i.e., four bays per section). Within a selected bay, eight spots (samples) were sampled from an area adjacent to a drinker and/or feeder area, and/or from the centre of the shed which were also selected via random numbers. These eight samples were mixed, and quartered to form a single composited sample for the relevant section, e.g., brooder 1. The same was done for brooder 2, grow-out 1, and grow-out 2, resulting in four main samples per shed (from a total of 32 litter samples per shed), representing the four main sections. Two chickens were collected in the region of the bays selected for sampling of litter resulting in eight chickens per section (e.g., brooder 1). These eight chickens were aseptically dissected on site to remove the ceca, which were then composited to form a single sample for that section (e.g., brooder 1); thus, a total of 32 chickens (samples) were collected per shed representing the four main sections. Final composited samples of litter and chicken were stored chilled. Litter was sampled on arrival at the laboratory; ceca were sampled within 22 hours of sample collection. Litter pH was measured as in Chinivasagam et al. (2012).

### Enumeration and Characterization of *Campylobacter*

Twenty-five grams of litter or ceca were weighed into 225 mL of Preston broth without antibiotics (Nutrient broth No2 with 5% lysed horse blood). The ceca were stomached (Smasher AESAPI064) and the litter was blended using a stick blender (barmix) for 1 minute. *Campylobacter* levels in litter were determined using a three-tube Most Probable Number (**MPN**) method as described by Chinivasagam et al. (2009). Serial dilutions of ceca were directly plated onto CCDA with selective supplement (Oxoid SR0155) and incubated at 37°C for 48 h under micro-aerobic conditions using Campygen (Oxoid, CN0025A). For both litter and ceca, three to five typical greyish, shiny, moist colonies representative of *Campylobacter* were randomly picked from the appropriate or countable CCDA plate and streaked onto Abeyta-Hunt-Bark agar as in Chinivasagam et al. (2009) for confirmation. DNA extracts were prepared by re-suspending a loopful of growth in 100 μl of sterile distilled water and heating at 95°C for 30 min. The suspension was cooled, centrifuged (at 14,000 × *g* for 45 s) and stored (at –18°C). The isolates were confirmed as *Campylobacter* spp. by a real-time PCR (Best et al., 2003) following which *Campylobacter* levels were presented as MPN/g for litter and CFU/g for ceca. The minimum detection for litter was log 0.6 MPN/g and for ceca log 2.0 CFU/g. The *Campylobacter* isolates were confirmed as *C. jejuni* or *C. coli* using the real-time PCR described by Best et al. (2003). A total of 825 isolates, 611 from F2 and 214 from F1, were positively identified to the species level and were presented as percentage species distribution. *E. coli* levels were enumerated as described in Chinivasagam et al., 2009 using the same serial dilutions used for litter and ceca.

### Statistical Methodology

Prior to commencement of the trial, “shed effects” sampling of surface litter was carried out, comparing the three sheds on F1. Analysis of variance for water activity, moisture, pH, and temperature showed no notable impact of the shed environment (data not presented). The study sheds were found to be approximately equal, thus validating the study design adopted on F1 and F2.

The time-series nature of the data was taken into account by an analysis of variance of repeated measures (Rowell and Walters, 1976), via the AREPMEASURES procedure of (GenStat, 2013). This forms an approximate split-plot analysis of variance (split for time). The Greenhouse-Geisser epsilon estimates the degree of temporal autocorrelation, and adjusts the probability levels for this. Sheds (within sites) were taken as the experimental unit, as the litter treatments were applied at this level. End of shed (brooder/growing) was included as a split-plot design within sheds, with a second split in the analysis of variance for times. Observations recorded as ‘less than limit of detection’ (**LoD**) were included in the analyses as half the LoD. As the sites had differing management practices, sites were included as a factor of interest (rather than as a random or blocking effect). The 5% significance level (*P* = 0.05) is used throughout. The four-way interaction (sites by treatments by shed-ends by times) was not significant in all analyses, so was dropped from the model.

## RESULTS

At the commencement of each cycle, sampling for litter only was carried out on d 7 to 9 (data not shown). During the 2-year period of this trial across farms cycles and treatments, *Campylobacter* was detected in litter only once, on d 9, cycle 6, F1, in “new litter treatment” and only at low levels, log 1.0–3.0 MPN/g (i.e., in both brooder-end segments only, where the chickens were present) and not in the chicken-free grow-out end of the shed. The farm was run as free-range for the first time (Table 1), though the flock was never out on the range during that period. Thus, other than this single and low-level instance, *Campylobacter* was never detected early in any cycle on both farms.

At first thin-out and final pick-up (analyzed separately), the statistical analysis for both litter and ceca showed that the three-way interaction (treatments by farms by times) was highly significant (*P* < 0.01), indicating that the patterns of *Campylobacter* emergence/presence across time vary between the farms, treatment, cycles, and pick-ups. These differences over cycles and time were not related to the litter treatments even though the litter treatments remained the same across two independent farms. *Campylobacter* levels in ceca and litter across six farming cycles on both farms for the three litter treatments are presented in Table 2.

**Table 2.** *Campylobacter* levels in ceca^#^ (C) (log CFU/g) and litter* (L) (log MPN/g) during first thin-out and final pick-up on Farms 1 (F1, 2009–2010) and Farm 2 (F2, 2010–2011) in new, partial and full re-use litter treatments across six farming cycles (Cy1, Cy2, Cy3, Cy4, Cy5, Cy6).

	First thin-out											
	Cy1**		Cy2		Cy3		Cy4		Cy5		Cy6	
CECA	F1	F2	F1	F2	F1	F2	F1	F2	F1	F2	F1	F2
**day**	**27**	**28**	**28**	**28**	**28**	**29**	**27**	**35**	**28**	**32**	**26**	**28**
New	nd	nd^b^	nd	nd	nd	4.8^a^	nd	8.4	nd	nd^b^	9.4^a^	7.2^b^
Partial	2.5	8.5^a^	nd	nd	nd	nd^b^	nd	8.6	nd	6.0^b^	9.3^a^	8.8^a^
Full	2.8	nd^b^	nd	nd	nd	nd^b^	nd	8.0	nd	7.3^a^	nd^b^	8.0^a,b^
LITTER	F1	F2	F1	F2	F1	F2	F1	F2	F1	F2	F1	F2
New	nd	nd^b^	nd	nd	nd	3.0^a^	nd	5.1	nd	nd^b^	5.5^a^	5.1
Partial	nd	2.9^a^	nd	nd	nd	nd^b^	nd	5.3	nd	1.1^b^	5.5^a^	5.2
Full	nd	nd^b^	nd	nd	nd	nd^b^	nd	5.0	nd	3.2^a^	nd^b^	4.8
Final pick-up												
CECA	F1	F2	F1	F2	F1	F2	F1	F2	F1$^{\hat{\,}}$	F2	F1$^{\hat{\,}}$$^{\hat{\,}}$	F2
**day**	**51**	**48**	**49**	**51**	**50**	**49**	**52**	**50**	**42**	**50**	**41**	**45**
New	6.8	7.2^b^	8.1	nd	8.5	8.4^a,b^	8.0	7.6^a,b^	4.6^a^	8.6	9.0	8.5
Partial	6.7	9.0^a^	8.5	nd	8.3	9.0^a^	8.0	8.6^a^	nd^b^	8.7	8.9	8.8
Full	6.4	8.7^a^	8.9	nd	7.5	7.5^b^	8.4	6.6^b^	nd^b^	8.8	9.0	8.9
LITTER	F1	F2	F1	F2	F1	F2	F1	F2	F1	F2	F1	F2
New	0.8	3.8	4.2	nd	2.7^b^	5.5	4.1^a,b^	4.8	nd^b^	5.5^a^	4.9	5.8^a^
Partial	1.0	3.7	3.7	nd	2.2^b^	4.5	3.2^b^	4.9	3.0^a^	4.9^a^	4.3	5.2^a,b^
Full	0.7	3.4	4.4	nd	4.8^a^	5.7	5.2^a^	4.3	nd^b^	3.0^b^	4.6	4.2^b^

^#^Mean of C1, C2, from brooder end and C3, C4 from grow-out end of a shed of allocated treatment.

*Mean of L1, L2 from brooder end and L3, L4 from the grow out end of shed of allocated treatment.

**All treatments equal during C1 (cycle 1).

^∧^During cycle 5 Farm 1 was picked up early.

^∧∧^All three litter treatments were in place but run as a free range cycle.

nd, not detected(minimum detection - litter log 0.6 MPN/g and ceca log 2 CFU/g).

^a,b^Within farms, cycles and pickups - treatment means with different superscripts are significantly (*P* <** 0.05) different.

### *Campylobacter*, First Thin-Out: Ceca

First thin-out ranged from d 26 to 35 across both farms (Table 2). During Cy1, the three litter treatments were similar (all-new bedding) as it followed a full clean-out of litter across the three test sheds on each farms. Irrespective of this situation, during Cy1 on F2, *Campylobacter* was detected only in the shed allocated to partial re-use, (ceca, log 8.5 CFU/g). Similarly during Cy1 on F1, *Campylobacter* was detected in ceca (log 2.5 and 2.8 CFU/g) in the two sheds allocated to both re-use treatments but not detected in new litter. Thus, there were differences in the pattern of emergence after a full clean-out of litter (Table 2).

Subsequently on F1, through four sequential cycles and until Cy6, *Campylobacter* did not emerge across all three litter treatments (not detected in litter and ceca). This was in contrast to F2, when *Campylobacter* emerged either intermittently (Cy3 and 5) or throughout litter treatments (Cy4 and 6) during the rest of the trial (Table 2). More specifically on F1 (Cy6), *Campylobacter* was detected in ceca at high levels (log 9.4 and log 9.3 CFU/g) in both new and partial re-use but not in full re-use; the levels were not significantly different. When *Campylobacter* was present intermittently on F2 (Cy3), it was present only in new litter treatment (log 4.8 CFU/g), while during Cy5, it was present in both partial (log 6.0 CFU/g) and full re-use (log 7.3 CFU/g) litter treatments; these levels were not significantly different. In contrast, when *Campylobacter* was present through all three litter treatments, on F2 (Cy4,) the levels (in ceca) were also not significantly different (ranged log 8.0–8.6 CFU/g). On F2 (Cy6) *Campylobacter* was present across all litter treatments and both partial re-use (log 8.8 CFU/g) and full re-use (log 8.0 CFU/g) were not significantly different compared to the new (log 7.2 CFU/g, *P* < 0.05). Thus from an overall perspective the pattern of *Campylobacter* emergence (and levels) on both farms during first thin-out across each cycle did not show any consistent pattern attributed to the litter treatments.

### *Campylobacter*, Final Pick-up - Ceca

Final pick-up ranged from d 45 to 51 (F1 and F2), with the exception of early pick-up on F1 (Cy5 and Cy6) Table 2. Just prior to final pick-up, *Campylobacter* had emerged across the majority of the litter treatments and was generally present at high levels (log 7.5–9.0 CFU/g) on both farms with the exception of F1, Cy1 (log 6.4–6.8 CFU/g across all three sheds), Table 2. On F1, *Campylobacter* levels in ceca during cycles 2, 3, 4, and 6 were not significantly different (*P* < 0.05) across the three litter treatments and were also in the similar range, (Table 2). The final cycle (Cy6) on F1 was run as a free-range cycle with high *Campylobacter* levels (log 9.0, 8.9, 9.0 CFU/g) the levels not significantly different, (Table 2). It was interesting to note that on F2, Cy2 remained a “*Campylobacter*-free cycle” across all three litter treatments (during both final pick-up and thin-out) with the organism never detected in either ceca or litter. *Campylobacter* was present across the rest of the cycles on F2, with levels during cycles 5 and 6 not significantly different (i.e., Cy5 log 8.6 to log 8.8 CFU/g and Cy6 log 8.5 to log 8.9 CFU/g) across all litter treatments (Table 2).

Thus, among the total of 10 cycles across both farms (total 12 but exclusive of Cy1), *Campylobacter* levels were significantly different (*P* < 0.05) across litter treatments only three times (two from F2 and one from F1), (Table 2). On F1, Cy5 low *Campylobacter* levels were detected prior to final pick-up only in new litter treatment (log 4.6) and below detection in both partial and full re-use litter treatments which can be attributed to an earlier than normal commercial final pick-up (d 41). Thus, as with the results for first thin-out, during final pick-up the differences between litter treatments were not related to the influence of any particular litter treatment, across both cycles and farms.

### *Campylobacter*, First Thin-Out and Final Pick-Up: Litter

*Campylobacter* levels in litter were generally four logs lower than the levels in ceca and the presence/absence of the organism in litter was always linked to ceca except on one occasion (Table 2). On F1, Cy1 the cecal *Campylobacter* levels were only low (log 2.5–2.8 MPN/g), a possible reason to be non-detectable in litter during first thin-out. The litter – ceca link could also be further demonstrated during Cy2, on F2, the “*Campylobacter*-free’ cycle when the organism was neither detected in litter nor in ceca. When comparing litter treatments and cycles, *Campylobacter* levels on F1 were not significantly different between litter treatments during three cycles (Cy1, 2, 6) and four sequential cycles (Cy1, 2, 3, 4) on F2 during final pick-up.

Interestingly, as the cycles on F2 progressed and during Cy5 (final pick-up), there tended to be a reduction in *Campylobacter* levels in full re-use litter (log 3.0 MPN/g) that was significantly lower (different *P* < 0.05) than both new (log 5.5 MPN/g) and partial re-use litter (log 4.9 MPN/g) Table 2. The reduction in full re-use continued to be observed on F2 (Cy6) though the *Campylobacter* levels in full re-use litter (log 4.2 MPN/g), were not significantly different to partial re-use (log 5.2 MPN/g) but significantly different to new (log 5.8 MPN/g, *P* < 0.05) Table 2. Irrespective of this reduction in *Campylobacter* levels in full-reuse litter, this pattern was not reflected in the ceca during those two cycles, suggesting that the litter treatment was not having an impact on *Campylobacter* levels in ceca.

On F1, two cycles (Cy3 and 4) presented variations across litter treatments during final pick-up, but these variations were not uniform. For example during Cy3, *Campylobacter* levels in both new (log 2.7 MPN/g) and partial re-use (log 2.2 MPN/g) were significantly different to full re-use litter (log 4.8 MPN/g, *P* < 0.05). A similar situation was observed during Cy4, F1, with the exception that full-re-use was not significantly different to new litter. As was for ceca, Cy5, F1 was the only cycle during which *Campylobacter* was not detected across two litter treatments (new and full re-use) which could be attributed to the early final pick-up on that farm at d 41, (Table 2).

### *E. coli*, First Thin-Out and Final Pick-Up: Ceca

Table 3 lists the mean *E. coli* levels in ceca. *E. coli* levels in both litter (not presented) and ceca did not vary much across treatments and cycles. In litter the highest *E. coli* levels (log 8.0 CFU/g) were generally evident around d 7 (data not shown) at the brooder ends, when young chicks are present. Unlike *Campylobacter*, *E. coli* was widely present across both thin-out and final pick-up, with not much variation between levels. From an overall perspective *E. coli* levels remained high and ranged from log 7.0–8.0 CFU/g. Just prior to first thin-out, there were no significant differences in *E. coli* levels in ceca between litter treatments across five cycles on both F1 (Cy1, 3, 4, 5, 6) and F2 (Cy1, 2, 4, 5, 6). Similarly just prior to final pick-up, *E. coli* levels in ceca were not significantly different between litter treatments across five cycles (Cy 1, 3, 4, 5, 6) on F1 and four cycles on F2 (Cy1, 2, 3, 4). There were some significant differences in Cy2 and 3. On F1 (Cy2) *E. coli* levels of both full re-use (log 7.9 CFU/g) and new (log 8.2 CFU/g) were significantly different (*P* < 0.05) from partial re-use (log 6.5 CFU/g). In contrast, during Cy3 on F2 partial (log 7.9 CFU/g) and full re-use (log 8.0 CFU/g) levels were significantly lower than in new (log 8.6 CFU/g, *P* < 0.05).

**Table 3.** *E. coli* levels in ceca^#^ (C) (log CFU/g) during first thin-out and final pick-up on Farms 1 (F1, 2009–2010) and Farm 2 (F2, 2010–2011) in new, partial and full re-use litter treatments across six farming cycles (Cy1, Cy2, Cy3, Cy4, Cy5, Cy6).

	Cy1**		Cy2		Cy3		Cy4		Cy5		Cy6	
	First thin-out											
day	27	28	28	28	28	29	27	35	28	32	26	28
	F1	F2	F1	F2	F1	F2	F1	F2	F1	F2	F1	F2
New	8.3	7.6	8.2^a^	8.3	7.9	8.6^a^	8.1	7.8	8.7	8.0	7.8	8.4
Partial	8.4	8.0	6.5^b^	8.0	7.8	7.9^b^	8.1	8.0	8.3	7.8	8.2	7.8
Full	8.1	7.9	7.9^a^	8.0	8.1	8.0^a,b^	7.9	7.9	8.3	7.8	8.4	7.9
	Final pick-up											
	F1	F2	F1	F2	F1	F2	F1	F2	F1	F2	F1	F2
**day**	**51**	**48**	**49**	**51**	**50**	**49**	**52**	**50**	**42**	**50**	**41**	**45**
New	7.1	7.3	6.9^a^	7.1	7.6	7.5	7.9	7.0	7.9	7.0^b^	8.0	7.7^a^
Partial	7.6	7.3	6.0^b^	6.9	7.3	7.1	8.2	7.2	7.9	6.9^b^	7.8	7.5^a^
Full	7.3	7.2	7.4^a^	6.8	7.0	7.5	8.3	7.4	7.9	7.6^a^	7.8	6.9^b^

^#^Mean of C1, C2, from brooder end and C3, C4 from grow-out end of a shed of allocated treatment.

**All treatments equal during C1 (cycle 1).

^a,b^Within farms, cycles and pickups - treatment means with different superscripts are significantly (*P* <** 0.05) different.

### Overall Effects of Litter Treatments on *Campylobacter* and *E. coli*

Tables 2 and 3 show varying patterns over time for the responses to the litter treatments. This indicates that, compared to the degree of variation within each shed, the six individual sheds are effectively following different time-paths (both within and across cycles, and between the farms). The overall main effects of the litter treatments (i.e., averaged across farms and times) were not significant, as shown in Table 4.

**Table 4.** Overall main effects of the litter treatments (i.e., averaged across farms and times).

	*P*-level for litter trt.	Full	New	Partial	Standard error
*Campylobacter* in ceca	0.46	5.15	5.43	5.90	0.349
*Campylobacter* in litter	0.11	2.30	2.60	2.58	0.057
*E. coli* in ceca	0.13	7.73	7.77	7.61	0.032

### *Campylobacter* Species Dominance Across Cycles

**Figure 1.**
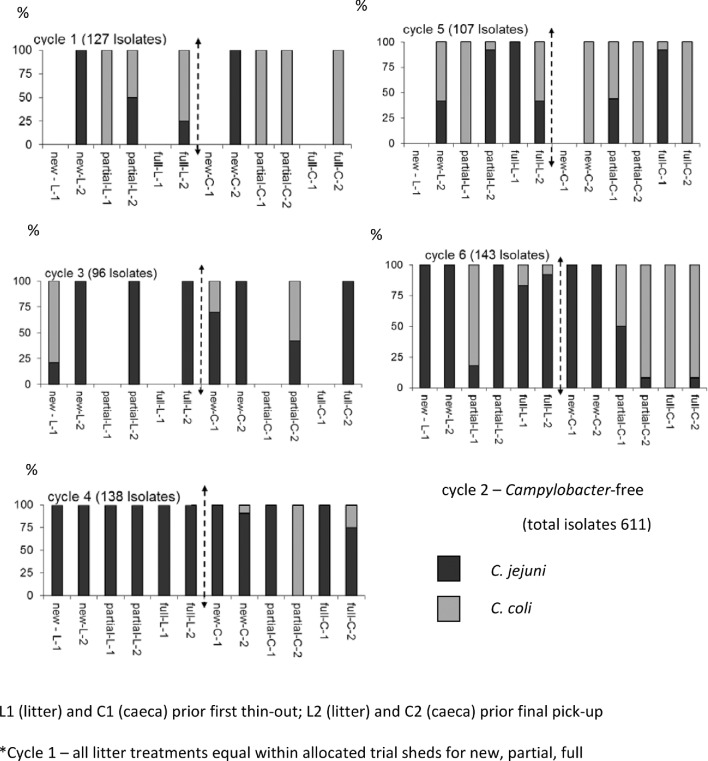
*Campylobacter* species distribution across cycles* 1 through 6 on Farm 2 for new, partial, and full re-use prior first thin-out in litter (L1) and ceca (C1) and final pick-up, litter (L2) and ceca (C2).

Table 5 presents a summary of the *Campylobacter* species diversity combined across litter, ceca, and treatments for each cycle on F1. During cycle 1, *C. jejuni* was dominant (100%) with a gradual transition to *C. coli* as the cycles progressed towards the sixth cycle (84%). More detailed analysis of patterns on F2 (Figure 1) illustrates the percentage of *C. jejuni* or *C. coli* in litter and ceca across treatments (new, partial, full) and cycles prior to first thin-out and prior to pick-up. *C. coli* tended to be associated with ceca in e.g., Cy1, 5, 6, and *C. jejuni* tended to be associated with litter (e.g., Cy3, 4, 6). There were tendencies for change in species mix between first thin-out and final pick-up (e.g., *C. coli* to *C. jejuni* in litter; Cy3, new; Cy5, partial; Cy6 partial) and *C. jejuni* to *C. coli* in ceca (Cy4, partial; Cy5, full). Thus there was no clear pattern attributable to the litter treatments (and farms), with some cycles totally dominated by one single species both in litter and ceca (Cy4, *C. jejuni* and Cy5, *C. coli*).

**Table 5.** Percentage^#^ *Campylobacter* species distribution across *cycles 1 through 6 (except 5) on Farm 1.

	*C. jejuni*	*C. coli*
Cycle 1	100	0
Cycle 2	84	16
Cycle 3	63	37
Cycle 4	28	72
Cycle 6	16	84

Total isolates 201.

^#^Combined litter and ceca for new, partial and full re-use treatments.

*Cy1 – all litter treatments equal within allocated sheds on both farms; Cy5 – not done.

### pH Levels in Litter

The litter pH values were around 8.00 or above close to both pick-up events (Table 6). When comparing the full re-use litter treatment (which had more aged litter) with new (across both pick-ups) there were only five instances when the pH values of full re-use litter were significantly different to new (*P* < 0.05) and only one instance when partial re-use was significantly different to new (*P* < 0.05). These results demonstrate no great variations in litter pH across treatments.

**Table 6.** pH levels in litter across six farming cycles for new, partial, and full re-use on Farm A.

	Cy1**	Cy2	Cy3	Cy4	Cy5	Cy6
	First pick-up					
New	8.54	8.14^b^	8.34	7.90	8.24^b^	8.36^a^
Partial	8.46	8.29^a,b^	8.40	8.47	8.40^a,b^	8.35^a^
Full	8.42	8.44^a^	8.35	8.39	8.54^a^	8.08^b^
	Final pick-up					
New	8.52	8.34	7.70^b^	8.25^b^	8.64	8.25^b^
Partial	8.40	8.35	7.93^a,b^	8.35^a,b^	8.40	8.76^a^
Full	8.54	8.50	7.97^a,b^	8.52^a,b^	8.47	8.59^a^

**All treatments equal during C1 (cycle 1).

^a,b^Within farms, cycles and pickups - treatment means with different superscripts are significantly (*P* <** 0.05) different.

## DISCUSSION

There is an intimate relationship between the bird and its bedding due to the potential for chickens to ingest litter during the farming cycle. Due to bird excretion, litter can be a source of microbiota, including *Campylobacter*, and this can influence the colonization and development of cecal microbiota (Torok et al., 2009). The reciprocal effects between the microbiotas present in the litter and the intestines of broilers can result in fresh litter having more environmental bacteria and re-used litter having bacteria of intestinal origin (Cressman et al., 2010). More specifically, litter has shown to be a source of *C. jejuni* infection to artificially inoculated chicks reared under controlled conditions (Montrose et al., 1985). Alternatively, the lack of pathogens in re-used litter during farming has been attributed to the presence of flora that is actively involved in the composting of organic matter (Lu et al., 2003b). The present study has shown that on F2, as full-reuse litter aged with time, *Campylobacter* levels (in litter) were more than two log lower at times (range log 1.6–2.6 MPN/g) than in new and partial re-use litter.

The current study that lasted two years with two farms adopting three different litter treatments across six sequential farming cycles has shown that adopted litter treatments had no relationship with both the emergence and levels of *Campylobacter* and *E. coli* in the ceca*.* However, both *Campylobacter* and *E. coli* were widespread in litter and ceca right across the trial; a litter–ceca relationship was apparent also observed for litter and aerosols in commercial broiler sheds (Chinivasagam et al., 2009). *E. coli* appeared at a very early age of the bird and at high levels until final pick-up. In contrast, *Campylobacter* emerged more or less at a more mature age of the bird with high levels (similar to *E. coli*) across the litter treatments.

*E. coli* was always present both in litter and ceca on both farms across time irrespective of the different litter treatments. High levels of bacteria (1.5 × 10^6^ CFU/g.) have been isolated from the ceca of newly hatched chicks (up to 21 hours) even when no food was consumed (Shapiro and Sarles, 1949). However, as soon as the chicks consumed food, the coliform levels (all confirmed to be *E. coli*) increased up to 1.5 × 10^8^ CFU/g within a few hours. These levels were maintained at 10^7^–10^8^ CFU/g from 0–200 d of age (Shapiro and Sarles, 1949) demonstrating the close link between *E. coli* with the bird right from the start. The ceca of chicken are also associated with various other microbes and can contain up to 10^10^–10^11^ cells/g of microorganisms within the cecal digesta (Gong et al., 2002). Thus the high levels of *E. coli* in litter in the present study are more a feature of bird excretion.

In the present study the levels of *E. coli* (for all the three litter treatments) were approximately log 7.0 CFU/g and ∼log 6.0 CFU/g in ceca and litter respectively with the cecal levels generally being a log higher than in litter. One of the key observations on both farms was the simultaneous uniform distribution of *E. coli* in both ceca and litter across the brooder and grow-out end sections (i.e., sections 1–4) throughout the six cycles for all three litter treatments. The uniform *E. coli* levels across brooder and grow-out sections appear to be a result of bird excretion and not as result of the build-up of *E. coli* levels across sequential re-use cycles where litter is treated by pile-up between re-use. *E. coli* is widely distributed within the shed (litter and aerosols) (Chinivasagam et al., 2009) and piling of litter between farming cycles has been shown to kill *E. coli* (Chinivasagam, 2009). It was therefore not surprising to observe in the current study that on d 7, *E. coli* was either absent or detected only in low levels in spread litter within the chicken-free brooding area (data not shown).

Unlike *E. coli, Campylobacter* was detected later in the production cycle of all commercial broiler batches monitored on both farms (except cycle 2 on F2 which was “*Campylobacter* free”). There was only one occasion where *Campylobacter* was detected at a very early stage of the production cycle, at d 9 in a free-range shed cycle of F1 with new litter and only in the both segments of the brooder end of that shed and (not the chicken-free grow-out end). Where both litter and ceca were collected from the same shed at the same time, litter was always shown to be positive when ceca was positive. Thus, even if the ceca was not tested at this stage, it may be possible that the chickens were *Campylobacter-*positive at the time. It is not possible that the free-range cycle was contributory, as the chicks did not have had access to the range. It also is unlikely that *Campylobacter* appearance was a carryover from a previous cycle, as it was not detected in spread litter following pile-up in the chicken-free brooder end at d 9 (data not shown). The organism has been shown to die-off in piled litter between partial re-use cycles (Chinivasagam, 2009). While the exact reason is uncertain, Cox et al. (2012) comprehensively reviewed the possibilities that can contribute to the early emergence of the organism, and thus it may not be unusual for flocks to be *Campylobacter*-positive at this early age, although vertical transmission of the organism remains controversial (Cox et al., 2012).

One of the interesting observations in the present study is the striking similarity of “uniform high levels” of *Campylobacter* and *E. coli* in the ceca (log 7.0 to log 8.0 CFU/g), and the uniform pattern of their distribution in both farms across the sheds brooder and grow-out areas (sections 1–4), litter treatments and cycles. *Campylobacter* and *E. coli* can be associated with the bird at different stages of the bird's life cycle. *Campylobacter* a commensal in poultry (Park, 2002), rapidly replicates in the mucous lining of cecal epithelial cells (Van Deun et al., 2008) and is closely associated with the intestinal mucosa and the crypts of the intestinal epithelium (Park, 2002), as *E. coli* which is associated with the cecal mucosa (Gong et al., 2002). During the present set of trials the cecal levels of *Campylobacter* were around log 8.0 CFU/g or even higher at times and has been previously reported (Daczkowska-Kozon et al., 2010). Chicken microbiota plays a major role in the “colonization resistance” of bacterial “pathogens” in the chicken gut (Chambers and Gong, 2011). The succession of gut microflora varies with bird age (Barnes, 1972; Apajalahti et al., 2001) possibly contributing to *Campylobacter* emerging mid-cycle and *E. coli* very early in the cycle.

The fact that *Campylobacter* assumed dominance at almost similar periods (prior both pick-ups) of the bird age (and similar levels) across 11 of the 12 cycles tested may suggest this pattern of dominance may be linked with the microbial succession (or ecology) prevalent in the ceca at the time. A succession of cecal microbial communities from one of transient flora to flora of increased complexity has been shown with bird age (i.e., d 14–28 and 49) (Lu et al., 2003a). Factors such as diet changes (i.e., starter, grower, finisher, and withdrawal) that occur routinely in commercial broilers, can influence the cecal bacterial community structure at various points of the cycle (Apajalahti et al., 2001) which in turn can contribute to *Campylobacter* proliferation. Thus external risk factors such as first pick-up or origin from sources external to the shed (Allen et al., 2008) seem not to be major drivers of the pattern and emergence of *Campylobacter* as observed in the current study.

During the present study there was a single cycle (cycle 2) on F2 that was “*Campylobacter* free” during the entire cycle across all three different litter treatments (three different sheds). This situation seemed unusual and interesting from this perspective that the rest of the five cycles on this farm had appreciable levels of *Campylobacter* prior to final pick-up across all three litter treatments and nothing varied during cycle 2. While several factors are linked as sources for flock colonization (Bull et al., 2006), there are limited studies on the reasons for a lack of colonization of a flock. Factors such as diet (Fernandez et al., 2000) immune status of the host and environmental conditions in the production system are said to play a role in *Campylobacter* colonization (Sahin et al., 2002). On-farm biosecurity (Allen et al., 2011) can prevent *Campylobacter* colonization though biosecurity was already an integral part of the current trial across all the rest of the cycles on both farms as was in cycle 2.

While the colonization dose may vary, the dose for colonization of 50% of the flock (or CD_50_) has been estimated to be 524 CFU. Once infected, *Campylobacter* can transmit via coprophagia (Line et al., 2008) and subsequent transmission rates within the shed will vary according to the commercial farming conditions and/or differing litter treatments. While innate immune responses can alter the host microbe interaction and has been attributed to colonization resistance between both resistant and susceptible birds (Connell et al., 2012) this alone does not fully explain why three sheds with different litter treatments were *Campylobacter* negative during an entire cycle of 51 d. The Cobb birds present across the three *Campylobacter*-free sheds during cycle 2 were from multiple parent stock without any clear link to the status of the parent flocks. A recent study by Gormley et al. (2014) has shown that both the chicken growth rate and breed did not contribute to a higher risk. These outcomes further emphasize the complexities involved in understanding *Campylobacter* colonization of flock, an area that requires further research.

Both *C. jejuni* and *C. coli* were widely distributed across both cycles and farms but their pattern of distribution could not be attributed to any litter treatment. On F1 there was a gradual transition of *C. jejuni* to *C. coli* (ceca and litter combined) across the three treatments and cycles. On F2 a more detailed analysis between litter and ceca presented a more complex pattern with both species represented. In the majority of the instances there were differences between litter and ceca but on other instances the species diversity matched. The disappearance of *C. jejuni* in organic flock coincided with the appearance of both bacteriophages and *C. coli* as the dominant strain (El-Shibiny et al., 2005). Host immunity or host mediated changes in the gut flora have also been suggested as possible contributory factors for initial *C. coli* and *C. jejuni* co-colonization and the subsequent displacement of the established *C. jejuni* in broilers (El-Shibiny et al., 2007). It is thus possible that both the ceca and the litter environments may have had a role in the complex *C. jejuni*–*C. coli* interactions in the cecum (and thus litter).

The present study has also demonstrated that *Campylobacter* had a poor survival potential in litter. Unlike *E. coli*, the *Campylobacter* levels in litter were lower than its levels in ceca. *Campylobacter* is a fragile organism (Klancnik et al., 2009) and characterized by rapid die-off in litter (Chinivasagam, 2009). *Campylobacter* does not survive well outside the host and does not have the many key regulators of stress defense (such as oxidative and osmoprotection stationary phase response) that are present in both *E. coli* and *Salmonella* (Park, 2002). The fact that it is a poor survivor outside and yet had the potential to reach these uniformly distributed high levels (log 8.0 to log 9.0 CFU/g in ceca) across the three litter treatments suggests that the microorganism's emergence can be closely linked to a complex set of factors within the cecum, rather than the litter practices adopted.

In conclusion, these studies carried out under commercial farming conditions over a 2-year period have demonstrated that re-use of litter did not directly influence either the timing of emergence or levels of bacterial concentration achieved (*Campylobacter* and *E. coli*) or a predominance of a *Campylobacter* species detected when compared to the use of new bedding. The factors that influence the above are complex and not directly a feature of the litter re-use practices adopted.
